# Genetic polymorphisms and susceptibility to lung disease

**DOI:** 10.1186/1477-5751-5-5

**Published:** 2006-04-11

**Authors:** Pauline L Lee, Carol West, Karen Crain, Lei Wang

**Affiliations:** 1 The Scripps Research Institute, Department of Molecular and Experimental Medicine, 10550 North Torrey Pines Road, La Jolla, 92037, USA

## Abstract

Susceptibility to infection by bacterium such as *Bacillus anthracis *has a genetic basis in mice and may also have a genetic basis in humans. In the limited human cases of inhalation anthrax, studies suggest that not all individuals exposed to anthrax spores were infected, but rather, individuals with underlying lung disease, particularly asthma, sarcoidosis and tuberculosis, might be more susceptible. In this study, we determined if polymorphisms in genes important in innate immunity are associated with increased susceptibility to infectious and non-infectious lung diseases, particularly tuberculosis and sarcoidosis, respectively, and therefore might be a risk factor for inhalation anthrax. Examination of 45 non-synonymous polymorphisms in ten genes: *p47phox *(*NCF1*), *p67phox *(*NCF2*), *p40phox *(*NCF4*), *p22phox *(*CYBA*), *gp91phox *(*CYBB*), *DUOX1*, *DUOX2*, *TLR2*, *TLR9 *and alpha 1-antitrypsin (*AAT*) in a cohort of 95 lung disease individuals and 95 control individuals did not show an association of these polymorphisms with increased susceptibility to lung disease.

## Introduction

Since October 2001, when *Bacillus anthracis *was released in the United States as an act of bioterrorism, there has been a greater interest in determining if there are risk factors for inhalation anthrax infection. Exposure to *Bacillus anthracis *spores does not cause infection in all exposed individuals [1]. Epidemiologic studies of individuals infected by inhalation anthrax have suggested that a weakened immune system might increase susceptibility to infection by *Bacillus anthracis *[2]. Some of the infected individuals had a history of chronic pulmonary disease, including asthma, sarcoidosis, and tuberculosis [2-4]. Studies in mice have demonstrated a genetic basis for anthrax sensitivity [5,6]. For example, macrophages from C3H mice are 100,000 times more sensitive to the *Bacillus anthracis *toxin than macrophages from A/J mice [6]. The current study examines whether there are genetic polymorphisms in humans associated with increased susceptibility to lung disease. Identification of genes associated with an increased risk of lung disease might identify individuals who might also be of increased susceptibility to inhalation anthrax infection.

The **N**AD(P)H **ox**idases (NOX) are a family of enzymes that are essential in host defense against microbial infection, as reviewed by Quinn and Gauss [7]. The central enzyme of the NAD(P)H oxidase is a flavin and heme-containing protein, the most well known being the phagocytic gp91phox (CYBB, NOX2) protein. gp91phox, and a number of related proteins including DUOX1 and DUOX2, are transmembrane proteins which transport electrons and generate reactive oxygen species (ROS) at the expense of NADH or NADPH. The activity of the oxidases are highly regulated by accessory proteins, including p22phox (CYBA), p47phox (NOXO1, NCF1), p67phox (NOXA2, NCF2), and p40phox (NCF4). Chronic Granulomatous Disease (CGD), associated with severe, recurrent, and chronic non-specific bacterial and fungal infections, is most commonly caused by mutations in *p47phox*, *gp91phox*, *p67phox*, and *p22phox *that severely compromise the respiratory burst activity of neutrophils.

Görlach et al were the first to identify the presence of at least one pseudogene copy of the *p47phox *(*NCF1*) gene on chromosome 7q11.23 [8]. By construction of a detailed physical map of this region Hockenhull et al determined that there were one normal wildtype copy and two pseudogene copies of *NCF1 *per chromosome [9]. Heyworth et al elegantly demonstrated that in some individuals, one of the pseudogene copies of *NCF1*, possibly by recombination or gene conversion, has reverted to the normal wildtype GTGT sequence (i.e. pseudowildtype) [10]. Thus, individuals with this low frequency polymorphism of *NCF1*, have 2 "wildtype" copies and one pseudogene copy per chromosome [10]. Therefore, individuals (with 2 chromosomes) can have a *NCF1 *pseudogene: wt copy ratio of either 2:1, 1:1 or 1:2. Although two groups have examined the association of the minor 1:1 and 1:2 alleles with inflammatory bowel disease, the conclusions were in conflict primarily due to differences in allele frequencies of the control population and sample size [11,12]. Other polymorphisms in *p47phox*, *p67phox *and *gp91phox*, have not been shown to be associated with human disease other than CGD. Recently *p47phox *has been shown by positional cloning to regulate the severity of arthritis in rats [13]. The H72Y polymorphisms in *p22phox *(*CYBA*), associated with reduced respiratory burst in isolated human neutrophils [14], but has yet to be shown to be clearly associated with a disease phenotype [15-17]. DUOX1 and DUOX2, which are expressed in lung epithelium, regulates H_2_O_2 _[18-20] and acid [21] production in the airway but have not been shown to be associated with lung disease. Mutations in *DUOX2 *have been shown to be associated with mild hypothyroidism [22-24].

TLR2 is the receptor for peptidoglycans, lipoteichoic acid, lipoarabinomannan, mycolylarabinogalactan, and zymosan. Anthrax infection is thought to be partially mediated through the TLR2 pathway since TLR2 deficient mice are resistant to infection by the Sterne strain of *Bacillus anthracis *and HEK293 cells expressing TLR2, but not TLR4, are able to signal in response to exposure to heat-inactivated *Bacillus anthracis *[25]. Inactivation and killing of the tuberculosis mycobacterium is also mediated through TLR2 since macrophages from Tlr2-deficient mice or human macrophages blocked by anti-TLR2 antibodies failed to kill the bacteria [26]. Tlr9 and Tlr2 double knockout mice display a more pronounced susceptibility to infection by tuberculosis than single gene knockout mice [27]. The *TLR2 *polymorphism R753Q [28] and the R677W polymorphism in humans [29-31] have been shown to be associated with increase risk for tuberculosis infection. The R753Q polymorphism was not associated with a generalized increased risk of infection, e.g. individuals with R753Q were less responsive to infection by *Borrelia burgdorferi*, which causes Lyme Disease [32] and R753Q was not associated with increased susceptibility to *Staphylococcus aureus *infection [33].

Alpha-1-anti-trypsin (AAT) deficiency has been associated with increased susceptibility to lung disease, particularly emphysema [34,35]. Although more than 70 variants have been described, only a few are associated with reduced AAT protein expression and/or reduced activity [35]. Several studies have suggested that simple heterozygosity for mutant alleles of *AAT *may predispose individuals to chronic obstructive lung disease [35-37]. The Z allele (E366K), which occurs at an allele frequency of 0.01–0.02 in people of European origin, is the most common allele associated with an increased risk of environmentally induced emphysema [34,38-40]. Homozygous individuals of the *AAT *S allele (E288V) are not at risk for emphysema but compound heterozygotes of the Z and S allele or a null allele are of increased risk [39,41]. Carriers of the AAT S and Z alleles are over-represented in individuals with lung cancer [42]

In this study, we attempted to determine whether normal nonsynonymous genetic variations identified by the Genbank SNP database or previously described in the literature to be present in the normal population in the genes for *p47phox *(*NCF1*), *p67phox *(*NCF2*), *p40phox *(*NCF4*), *gp91phox *(*CYBB*), *p22phox *(*CYBA*), *DUOX1*, *DUOX2*, *TLR2*, *TLR9 *and alpha-1 anti-trypsin (*AAT*) are associated with an increased susceptibility to tuberculosis, sarcoidosis, recurrent pneumonia, and atypical mycobacterial infection.

## Materials and methods

### Study participants

Anonymized blood samples from control individuals of European, non-Hispanic origin (n = 95) were obtained from Kaiser Permanente [43] or from The Scripps Research Institute GCRC blood drawing program. From a group of 31,247 participants in a Kaiser Permanente study of European, non-Hispanic origin [43], all individuals that had a documented medical history with hospitalization for lung diseases: atypical mycobacterial infection (n = 1), repeated episodes of pneumonia (n = 5), sarcoidosis (n = 46), and tuberculosis (n = 43), were selected and will be referred to as the lung disease group (n = 95). The participants in the Kaiser Permanente study were members of Kaiser Permanente attending a Health Appraisal Clinic and were not selected for underlying acute or chronic disease. All human samples were obtained with written consent. Approvals for the protocols involving the use of human individuals were obtained from the institutional review boards of The Scripps Research Institute and Kaiser Permanente.

### p47phox/NCF1 pseudogene: wildtype ratio

Amplification of the region of p47phox exon 2 with the wildtype GTGT sequence and the pseudogene delGT sequence were amplified using primers p47phox/NCF1 Ex2F GCTTCCTCCAGTGGGTAGTGGGATC and p47phox/NCF 161R GGAACTCGTAGATCTCGGTGAAGC and ^32^P-labeled p47phox/NCF1 Ex2F primer under standard PCR conditions for 25 cycles. The ^32^P-labeled amplified DNA products were separated on a 10% acrylamide/urea/TBE sequencing gel. Autoradiography was used to visualize the wildtype and pseudogene amplified products, which differ by 2 nucleotides in length.

### Genotyping of single nucleotide polymorphisms (SNPs) by allele specific oligomer hybridization (ASOH)

For the genes of this study, non-synonymous SNPs identified in Genbank's SNP database and/or non-synonynous SNPs associated with lung disease were investigated. Amplification of DNA regions encompassing the SNPs were amplified using the primers listed in Table 1. ASOH was performed using standard hybridization conditions [44] using ^32^P radiolabeled probes and washing temperatures described in Table 1. Genotyping was determined following visualization of the hybridized probe by autoradiography.

**Table 1:** Primer List. List of primers used for DNA amplification and ASOH.

**Primer name**	**Sequence**	**Temp °C**
p47 Ex2F	GCTTCCTCCAGTGGGTAGTGGGATC	60
p47 161R	GGAACTCGTAGATCTCGGTGAAGC	
**Primer name**	**Sequence**	**Temp °C**

p40 Ex2F	GTGCTGAGAGACGAATGTTGG	60
p40 Ex2R	GGGCAAGGTTCAGAGGTCAG	
p40 Ex5F	GACGGGACATCTAGGCTGG	60
p40 Ex5R	GGCTCTGGCCATGTGGAAG	
p40 Ex8F	TCTGAGGCGTGGCTCTGCTG	60
p40 Ex8R	GCTCATCTGGGAGCCACTGG	
p40 Ex10F	ATGACACGGGCTTGTATCAGG	60
p40 Ex10R	GAGCTGAAGGTTTTTGCTGGTG	
p40 86T	TGCTGACATCGAGGAGA	53
p40 86C	TGCTGACACCGAGGAGA	53
p40 353G	CCTGCTCAGCCTGCCGG	62
p40 353A	CCTGCTCAACCTGCCGG	61
p40 815C	ACGACCACCGCCCCTCA	58
p40 815T	ACGACCACTGCCCCTCA	56
p40 911C	GGACGTAGCGCTCATGG	57
p40 911A	GGACGTAGAGCTCATGG	55

**Primer name**	**Sequence**	**Temp °C**
p67 Ex3F	CTGGGCACCACAGGGAGCTA	58
p67 Ex3R	CACCAAGCCCGCAACACTGA	
p67 Ex6F	GGGCTTCTATGTGGTTATCTCAA	60
p67 Ex6R	CCACAAGGAGGCTACCCTCTTCT	
p67 Ex9F	GAGCCCAGGCAGGCTCAGTGTCAT	60
p67 Ex10R	GCCATCTCAAGGCGGGCTCAAGA	
p67 Ex11F	GTGTTTCCCCACATCCAC	60
p67 Ex11R	AAGGCAGGGAGAGGAACT	
p67 Ex13F	CAAGGGTTGGGCTAAAGGAC	60
p67 Ex14R	GTGTTCTCACACCACAGAGTCAG	
p67 542G	TGTGGGCAGGCTGTTTC	55
p67 542A	TGTGGGCAAGCTGTTTC	53
p67 836C	CTGGGCCACGGTCATGT	57
p67 836T	CTGGGCCATGGTCATGT	55
p67 983G	CCCTGGAAGACCCCAGC	47
p67 983A	CCCTGGAAAACCCCAGC	47
p67 1105G	CTCAGCCCGGGCTCCCC	50
p67 1105A	CTCAGCCCAGGCTCCCC	50
p67 1167C	GCTGGAACACACTAAGCTG	54
p67 1167A	GCTGGAACAAACTAAGCTG	54
p67 1183C	CCAGCTATCGGCCTCGG	57
p67 1183T	CCAGCTATTGGCCTCGG	57

**Primer name**	**Sequence**	**Temp °C**

p22 Ex 2F	GACCCTGTCACTGTGCTGTG	61
p22 Ex 2R	GAGGCAAACAGCTCACTGTG	
p22 Ex 3F	CTGAGCTGGGCTGTTCCTT	63
p22 Ex 3R	CCACCCAACCCTGTGAGC	
p22 Ex 4F	CAGCAAAGGAGTCCCGAGT	60
p22 Ex 4R	GGAAAAACACTGAGGTAAGT	
p22 Ex 5F	AAGGCTGAGAACACCCAGG	60
p22 Ex 5R	GCTCAGCCTACAGAGCCG	
p22 Ex 6F	GACCCAGGTCCTGGCTGTG	60+DMSO
p22 Ex 6R	AGGCTCACGCGCTCCCGG	
p22 85A	TCGTGGCCACAGCTGGG	59
p22 85G	TCGTGGCCGCAGCTGGG	59
p22 113T	GTGGTACTTTGGTGCCT	52
p22 113C	GTGGTACTCTGGTGCCT	52
p22 179A	GAAGAGGAAGAAGGGCT	51
p22 179C	GAAGAGGACGAAGGGCT	53
p22 214C	GACAGAAGCACATGACC	53
p22 214T	GACAGAAGTACATGACC	51
p22 403G	CGCCCATCGAGCCCAAG	59
p22 403A	CGCCCATCAAGCCCAAG	56
p22 521C	GCTGCGGCGGCGGCG	62
p22 521T	GCTGCGGTGGCGGCG	60

**Primer name**	**Sequence**	**Temp °C**
gp91phox Ex 9F	CTAAAGCAGAGATCTAAGTGG	61
gp91phox Ex 9R	ACGGTGACCACAGAAATAGCTACCT	
gp91phox Ex 11F	GTTTCTAGGCATTCTGAGCATCAAG	61
gp91phox Ex 11R	GTTCGTAAGCCCTGTACACTATG	
gp91phox Ex 12F	GTGCCTTGGTTAGAATAGCTTGTG	61
gp91phox Ex 12R	GTTGAAGATATCTGGAATCTTCTGTTG	
gp91phox 907C	TGGTCACTCACCCTTTC	50
gp91phox 907A	TGGTCACTAACCCTTTC	48
gp91phox 1414G	ACAATGCCGGCTTCCTC	55
gp91phox 1414A	ACAATGCCAGCTTCCTC	53
gp91phox 1499A	GGAGAAAGATGTGATC	48
gp91phox 1499G	GGAGAAAGGTGTGATC	50

**Primer name**	**Sequence**	**Temp °C**

DUOX1 27F	AGAGAGATCTCCTCTCAAGG	58
DUOX1 27R	GGTCACCGGAAGAGCTGAG	
DUOX1 28F	GGGACCTTGGAAGCTCCAG	58
DUOX1 28R	GGACGTCGAGAAGTGAAGAG	
DUOX1 3532T	GGTCTGAGTTCCCCCAG	58
DUOX1 3532C	GGTCTGAGCTCCCCCAG	60
DUOX1 3647G	GCCGCCGCCGCAGTTTCC	66
DUOX1 3647A	GCCGCCGCCACAGTTTCC	63

**Primer name**	**Sequence**	**Temp °C**
DUOX2 Ex5F	ATGTTCTTTCCGACGTGGTGAG	63
DUOX2 Ex6R	GCGCCGCCCACATGAGCAG	
DUOX2 Ex17F	GCCTGCTCAGACTCACAGAG	62
DUOX2 Ex17R	ACTCCTTAGGGATCTTGAGCAG	
DUOX2 Ex24F	GATGCCTGCCAGATCCCCAG	62
DUOX2 Ex25R	TGGCCGCCGTGCCTCGTG	
DUOX2 413T	TGGAGACCTCGTGTTCG	54
DUOX2 413C	TGGAGACCCCGTGTTCG	56
DUOX2 429A	CCGAACAGCGCGGGGAC	60
DUOX2 429C	CCGACCAGCGCGGGGAC	63
DUOX2 597-8GG	GCTTCTCGGGGGGACAG	58
DUOX2 597-8GA	GCTTCTCGAGGGGACAG	56
DUOX2 597-8CG	GCTTCTCCGGGGGACAG	58
DUOX2 597-8CA	GCTTCTCCAGGGGACAG	56
DUOX2 2048G	TGTGCTCCGTGTGGTCC	56
DUOX2 2048A	TGTGCTCCATGTGGTCC	54
DUOX2 3026G	CACTCCCCGGCTGTACA	56
DUOX2 3026A	CACTCCCCAGCTGTACA	52
DUOX2 3200T	CTTTGCCTTGCCACCCT	53
DUOX2 3200C	CTTTGCCTCGCCACCCT	55

**Primer name**	**Sequence**	**Temp °C**

TLR2 450F	ATTGCAAATCCTGAGAGTGG	58
TLR2 688R	GCAGTTCCAAACATTCCACG	
TLR2 1141F	GCCTGTGAGGATGCCTGG	60
TLR2 1827R	GCACAGGACCCCCGTGAG	
TLR2 1782F	GTGCTGTGCTCTGTTCCTG	60
TLR2 2392R	TCCCAACTAGACAAAGACTGG	
TLR2 170T	GAAAAGATTTTGCTGGAC	53
TLR2 170Tdel	GAAAAGATTTGCTGGAC	53
TLR2 1892C	GGAAGCCCAGGAAAGCT	55
TLR2 1892A	GGAAGCACAGGAAAGCT	53
TLR2 2258G	CAAGCTGCGGAAGATAA	50
TLR2 2258A	CAAGCTGCAGAAGATAA	48

**Primer name**	**Sequence**	**Temp °C**
TLR9 Ex2F	GTGGGTGGAGGTAGAGCTG	60
TLR9 365R	ACAGCCAAGAAGGTGCTGG	
TLR9 2501F	TGCTGCATCACCTCTGTGG	54
TLR9 2794R	TGCGGCTGCCATAGACCG	
TLR9 13C	GTTTCTGCCGCAGCGCC	60
TLR9 13T	GTTTCTGCTGCAGCGCC	58
TLR9 237T	CACCTCCATGATTCTGA	52
TLR9 237G	CACCTCCAGGATTCTGA	54
TLR9 296C	GAACTGCCCGCCGGTTG	58
TLR9 296T	GAACTGCCTGCCGGTTG	60
TLR9 2588G	AAGTGGGCGAGATGAGG	57
TLR9 2588A	AAGTGGGCAAGATGAGG	55
TLR9 2644G	CGCAGAGCGCAGTGGCA	60
TLR9 2644A	CGCAGAGCACAGTGGCA	58

**Primer name**	**Sequence**	**Temp °C**
AAT Ex2F	TGTCGGCAAGTACTTGGCACAG	60
AAT Ex2R	CATAATGCATTGCCAAGGAGAG	
AAT Ex3F	CAGATGATGAAGCTGAGCCTCG	65
AAT Ex3R	AGCCCTCTGGCCAGTCCTGATG	
AAT Ex5F	GAGCAAGGCCTATGTGACAGG	60
AAT Ex5R	AGCTCAACCCTTCTTTAATGTCAT	
AAT 374G	ACTCCTCCGTACCCTCA	56
AAT 374A	ACTCCTCCATACCCTCA	54
AAT 863A	GCACCTGGAAAATGAAC	50
AAT 863T	GCACCTGGTAAATGAAC	50
AAT 1096G	CCATCGACGAGAAAGGG	56
AAT 1096A	CCATCGACAAGAAAGGG	54

### Statistics

The Fisher's Exact test was performed with GraphPad InStat using the raw data entered into a 2 × 2 contingency table. Power calculations were performed to give the probability of finding the differences between the gene frequencies as statistically significant, given the sample size.

## Results

We examined 95 individuals of European, non-Hispanic origin with documented medical history with hospitalization for lung disease (46 with sarcoidosis, 43 with tuberculosis, five with recurrent pneumonia, and one with atypical mycobacterial infection) and 95 control individuals of European, non-Hispanic origin for differences in allele frequencies in genes involved in innate immunity.

### P47phox/(NCF1)

Examination of the pseudogene: wt copy ratio of control versus lung disease individuals demonstrated no statistically significant difference in the frequencies of the pseudogene: wt ratios in the lung disease group as compared to the control group (Table 2).

**Table 2:** Pseudogene versus gene ratio. p47phox/NCF1 pseudogene: wt gene ratio in lung disease and control individuals. The data are presented as number of individuals with the indicated pseudogene:wt ratio and the number within parentheses indicates the calculated frequency.

**p47phox/NCF1 (Pseudogene: wt)**	**control (n = 59)**	**Lung Disease (n = 64)**
2:1	46 (0.78)	51 (0.80)
1:1	13 (0.22)	12 (0.19)
1:2	0 (0)	1 (0.02)

### p67phox (NCF2), p40phox (NCF4), p22phox (CYBA), gp91phox (CYBB), DUOX1, DUOX2

SNPs in the *p67phox *(*NCF2*), *p40phox *(*NCF4*), *p22phox *(*CYBA*) and *gp91phox *(*CYBB*), *DUOX1 *and *DUOX2 *genes were examined. Some SNPs did not occur at a high enough frequency to be detected in our samples. None of the allele frequencies differed significantly between the lung disease and the control groups (Table 3).

**Table 3:** Summary of SNP Analyses. SNP analyses of candidate genes in lung disease versus control groups. Numbering of SNPs start from the ATG initiator methionine of the cDNA. Data are presented as number of alleles identified divided by total number of alleles examined. Numbers within parentheses are the calculated allele frequencies. Power calculations were performed using number of subjects.

**p67phox (NCF2)**	**dbSNP rs#**	**SNP**	**amino acid**	**Control**	**Lung Disease**	**Power to detect 2× increase**	**Power to detect 1.5× increase**
Exon 6	rs2274064	542 A/G	K181R	79/186 (0.43)	91/190 (0.48)	0.98	0.96
Exon 9	rs13306581	836 C/T	T279M	0	0		
Exon 11		983 G/A	R 328K	0	0		
Exon 13		1105 G/A	G369R	0	0		
Exon 13	rs17849502	1167 C/A	H389Q	12/190 (0.06)	10/188 (0.05)	0.22	
Exon 14	rs13306575	1183 C/T	R395W	0	0		

**p22phox (CYBA)**	**dbSNP rs#**	**SNP**	**amino acid**	**Control**	**Lung Disease**	**Power to detect 2× increase**	**Power to detect 1.5× increase**
Exon 2		85 A/G	T29A	0	0		
Exon 2		113 T/C	F38S	0	0		
Exon 3		179 A/C	K60S	4/190 (0.02)	0	0.06	
Exon 4	rs4673	214 C/T	H72Y	61/180 (0.34)	60/190 (0.37)	0.99	0.61
Exon 6		403 G/A	E135K	0	0		
Exon 6	rs17845095	521 C/T	A174V	93/176 (0.41)	88/190 (0.46)	0.99	0.79

**p40phox (NCF4)**	**dbSNP rs#**	**SNP**	**amino acid**	**Control**	**Lung Disease**	**Power to detect 2× increase**	**Power to detect 1.5× increase**
Exon 2	rs13057803	86 T/C	I29T	0	0		
Exon 5	rs9610595	353 G/A	S118N	0	0		
Exon 8		815 G/A	P272L	30/190 (0.16)	29/190 (0.15)	0.68	0.22
Exon 10	rs5995361	911 C/A	A304E	0	0		
**gp91phox (CYBB)**	**dbSNP rs#**	**SNP**	**amino acid**	**Control**	**Lung Disease**	**Power to detect 2× increase**	**Power to detect 1.5× increase**

Exon 9	rs28935182	907 C/A	H303N	0	0		
Exon 11	rs13306300	1414 G/A	G472S	0	0		
Exon 12	rs28935181	1499 A/G	D500G	0	0		

**Duox 1**	**dbSNP rs#**	**SNP**	**amino acid**	**Control**	**Lung Disease**	**Power to detect 2× increase**	**Power to detect 1.5× increase**
Exon 27	rs2458236	3532 T/C	F1178L	64/184 (0.35)	56/154 (0.36)	0.99	0.63
Exon 28	rs2292466	3647 G/A	R1216H	0	0		

**Duox 2**	**dbSNP rs#**	**SNP**	**amino acid**	**Control**	**Lung Disease**	**Power to detect 2× increase**	**Power to detect 1.5× increase**
Exon 5	rs2001616	413 C/T	P138L	26/188 (0.14)	22/190 (0.12)	0.59	
Exon 5	rs7166994	429 C/A	D143E	0	0		
Exon 6	rs2467827	598 G/A	G200R	1/188 (0.01)	1/190 (0.01)	0.05	
Exon 17	rs8028305	2048 G/A	R683H	0	0		
Exon 24	rs2277611	3026 G/A	A1009Q	0	0		
Exon 25	rs269868	3200 T/C	L1067S	22/186 (0.12)	15/190 (0.08)	0.5	
**TLR2**	**dbSNP rs#**	**SNP**	**amino acid**	**Control**	**Lung Disease**	**Power to detect 2× increase**	**Power to detect 1.5× increase**

Exon 2	rs3840097	510Tdel	F170Lfs	0	0		
Exon 2	rs5743699	1232C/T	T411I	nd	0		
Exon 2	rs5743702	1667T/C	I556T	nd	0		
Exon 2	rs5743703	1736G/A	R579H	nd	0		
Exon 2	rs5743704	1892C/A	P631H	9/184 (0.05)	8/188 (0.04)	0.18	
Exon 2		2029C/T	R677W	nd	0		
Exon 2	rs5743706	2143T/A	Y715N	nd	0		
Exon 2	rs5743707	2145T/G	Y715Stop	nd	0		
Exon 2	rs5743708	2258G/A	R753Q	2/182 (0.01)	4/188 (0.02)	0.05	
Exon 2		2304G/T	E768D	nd	0		

**TLR9**	**dbSNP rs#**	**SNP**	**amino acid**	**Control**	**Lung Disease**	**Power to detect 2× increase**	**Power to detect 1.5× increase**
Exon 2	rs5743842	13 C/T	R5C	2/190 (0.01)	0	0.05	
Exon 2	rs5743843	237T/G	H79Q	0	0		
Exon 2	rs5743844	296 C/T	P99L	0	0		
Exon 2	rs5743845	2588 G/A	R863Q	6/170 (0.04)	0/186 (0*)	0.14	
Exon 2	rs5743746	2644 G/A	A882T	0	0		
**AAT (SERPINA1)**	**dbSNP rs#**	**SNP**	**amino acid**	**Control**	**Lung Disease**	**Power to detect 2× increase**	**Power to detect 1.5× increase**

Exon 2	rs709932	374G/A	R125H	38/178 (0.21)	29/182 (0.16)	0.85	0.31
Exon 3	rs17580	863A/T	E288V	5/190 (0.03)	4/190 (0.02)	0.1	
Exon 4	rs28929474	1096G/A	E366K	4/192 (0.02)	2/190 (0.01)	0.07	

### TLR2, TLR9, AAT

*TLR2*, *TLR9*, and *AAT *genes were examined. Again, many SNPs did not occur at high enough frequency to be observed. Most of the allele frequencies did not differ between the lung disease and control groups. The *TLR2 *polymorphism R753Q, associated with tuberculosis, was not shown to be different between the control or lung disease group. The *TLR2 *R677W polymorphism, also associated with tuberculosis, was not observed in either group. The R863Q SNP in TLR9 was absent from the lung disease group indicating that this polymorphism was not associated with increased lung disease. The *AAT *S (Glu288Val) and Z (E366K) alleles, associated with chronic obstructive lung disease, were examined and there was no difference in allele frequencies between the control and lung disease groups (Table 3).

## Discussion

Since only a subset of individuals exposed to *Bacillus anthracis *spores develop pulmonary disease, the most life-threatening form of anthrax infection, it would be important to identify factors that lead to susceptibility to this type of infection. This might make it possible to identify those individuals who are at greatest risk and to provide them with the most aggressive treatment at the outset of infection. The ability to thus triage individuals in the case of a bioterrorism attack would be valuable. Moreover, understanding genetic susceptibility could lead to better management of individuals with pulmonary anthrax infection.

The genetic influences on resistance to infection are very strong. Indeed, genetic influences on resistance to infection appear to be greater than genetic influences on cancer or cardiovascular disease [45]. In the past few decades a considerable number of polymorphisms have been shown to cause infectious disease susceptibility in mice [6] and in humans [28,31,46]. Because infections caused by *Bacillus anthracis *are rare it was impossible to examine candidate polymorphisms in patients who actually developed pulmonary anthrax. Instead, it was necessary to use surrogate infections such as unusual mycobacterial infections, recurrent pneumonia, and tuberculosis or examine lung diseases such as sarcoidosis, which has been reported in cases of inhalation anthrax, for this study. The "lung disease group" in this study represented all the individuals with documented hospitalization for lung disease from a collection of 31,247 individuals of European, non-Hispanic origin unselected for any particular acute or chronic health problem. Candidate genes were chosen on the basis of their role in immunity against chronic infection as well as their participation in the innate immune response. This is a reasonable approach, since defects in the immune system generally increases susceptibility not to a single organism, but rather to multiple organisms that share some features in the pathogenesis of the disease that they produce.

Our analyses of genes of the NAD(P)H oxidase, *p47 *(*NCF1*), *p67phox *(*NCF2*), *p40phox *(*NCF4*), *p22phox *(*CYBA*), and *gp91phox *(*CYBB*), as well as other genes involved in innate immunity such as *DUOX1 *and *2*, *TLR2*, *TLR9 *and *AAT *demonstrated that there were no differences between the control and lung disease group comprised of primarily sarcoidosis and tuberculosis individuals. There may, of course, be many other polymorphisms that affect susceptibility to *Bacillus anthracis*. Although the genes that we chose seemed to be reasonable candidates; there are many additional genes encoding products that could be important in effecting the course of anthrax in humans. For example, it has been suggested that susceptibility to *Bacillus anthracis *might involve *myD88 *[25]. Furthermore, susceptibility to infection by tuberculosis may be altered by variations in the vitamin D receptor gene [47]. Similarly, sarcoidosis has been shown to be associated with particular alleles in *BTNL2 *[48,49], *IL18 *[50], and *IFNa *[51], and *SLC11A1 *[52].

## Competing interests

The author(s) declare that they have no competing interests.

## Authors' contributions

Each author contributed substantially to the design, acquisition, and analysis of the data. PLL supervised the project and wrote the manuscript. Each author has read and approved the manuscript prior to submission.
